# Cognitive Beliefs Across the Symptom Dimensions of Pediatric Obsessive-Compulsive Disorder: Type of Symptom Matters

**DOI:** 10.1016/j.beth.2021.08.001

**Published:** 2021-08-20

**Authors:** Matti Cervin, Morgan M. McNeel, Sabine Wilhelm, Joseph F. McGuire, Tanya K. Murphy, Brent J. Small, Daniel A. Geller, Eric A. Storch

**Affiliations:** Lund University; Baylor College of Medicine; Massachusetts General Hospital and Harvard Medical School; Johns Hopkins University School of Medicine; University of South Florida; University of South Florida; Massachusetts General Hospital and Harvard Medical School; Baylor College of Medicine

**Keywords:** OCD, symptom dimensions, cognitive beliefs, metacognitive, children, adolescents

## Abstract

The cognitive model of obsessive-compulsive disorder (OCD) posits that dysfunctional cognitive beliefs are crucial to the onset and maintenance of OCD; however, the relationship between these cognitive beliefs and the heterogeneity of OCD symptoms in children and adolescents remains unknown. We examined how the major belief domains of the cognitive model (inflated responsibility/threat estimation, perfectionism/intolerance of uncertainty, importance/control of thoughts) and dysfunctional metacognitions were related to OCD symptoms across the following dimensions: doubting/checking, obsessing, hoarding, washing, ordering, and neutralization. Self-report ratings from 137 treatment-seeking youth with OCD were analyzed. When cognitive beliefs and symptom dimensions were analyzed in tandem, inflated responsibility/threat estimation and dysfunctional metacognitions were uniquely related to doubting/checking, obsessing, and hoarding and perfectionism/intolerance of uncertainty to ordering. Cognitive beliefs explained a large proportion of variation in doubting/checking (61%) and obsessing (46%), but much less so in ordering (15%), hoarding (14%), neutralization (8%), and washing (3%). Similar relations between cognitive beliefs and symptom dimensions were present in children and adolescents. Cognitive beliefs appear to be relevant for pediatric OCD related to harm, responsibility, and checking, but they do not map clearly onto contamination and symmetry-related symptoms. Implications for OCD etiology and treatment are discussed.

Pediatric obsessive-compulsive disorder (OCD) is characterized by the presence of obsessions and/or compulsions (American Psychiatric Association, 2013). Most youth with OCD endorse numerous symptoms at any one point in time (Geller & March, 2012; Rettew et al., 1992). Symptoms may include compulsive hand washing, checking behaviors, repeating, counting and ordering rituals, collecting, magical thinking or rituals involving other people, as well as obsessions about contamination, aggression, hoarding, somatic concerns, superstitious beliefs and religious or sexual concerns (Scahill et al., 1997). These seemingly disconnected idiosyncratic symptoms can be conceptualized into overarching dimensions with an empirically supported four-factor model: (1) obsessions about harm/responsibility and checking compulsions, (2) hoarding obsessions and compulsions, (3) symmetry obsessions and arranging/ordering compulsions and (4) contamination obsessions and cleaning compulsions (Bloch et al., 2008). In addition to replicating these dimensions, recent work has outlined four others: body-focused symptoms, superstition, transformation fears, and loss/separation concerns (Cervin et al., 2021). While hoarding was classified as a discrete disorder in the DSM-5, it can also present as a part of OCD in the form of obsessions and compulsions (American Psychiatric Association, 2013). Symptom structures similar to those reported in Bloch et al. (2008) have been identified using pediatric samples, and among youth, these dimensions have been shown to be differentially associated with comorbidity patterns and sex distribution (Hojgaard et al., 2016).

The symptom dimensions of OCD are temporally stable and show differences in heritability, neural underpinnings, and neurocognitive functioning (Fernández de la Cruz et al., 2013; Leckman & Bloch, 2008; McGuire et al., 2014; Stewart et al., 2008). In youth, symptoms within the harm/responsibility/checking dimension have been shown to be largely motivated by fear and anxiety, making them similar to symptoms of anxiety disorders (Cervin et al., 2020b). In contrast, symptoms within the contamination and symmetry dimensions have been shown to be more strongly associated with disgust and “not just right” feelings, respectively, than with anxiety (Cervin et al., 2020a, 2020b). The notion that OCD may not be purely motivated by fear and anxiety was one of the reasons for the removal of OCD from the anxiety disorders chapter in DSM-5 (Van Ameringen et al., 2014), and it challenges many influential psychological models of OCD that all highlight the central role of fear and anxiety in the onset and maintenance of symptoms (Abramowitz et al., 2009; Rachman, 1997).

The cognitive model of OCD suggests that dysfunctional beliefs and maladaptive interpretations of intrusive thoughts play a significant role in the development and maintenance of the disorder (Rachman, 1997). This model is based on the tenet that intrusive thoughts are common in the general population and that the development of OCD stems from (mis)interpretation of naturally occurring cognitive phenomena. Metacognitive beliefs, or beliefs about one’s thinking and strategies to control cognitive processes, are increasingly studied in relation to psychopathology. It has been suggested that, alongside the more specific cognitive beliefs outlined in the cognitive model, metacognitive beliefs represent a key cognitive process in the development and maintenance of OCD (Fisher & Wells, 2008). Given the theoretical and statistical overlap among different cognitive and metacognitive belief domains, it is important to study them in tandem so that unique associations between cognitive processes and OCD can be outlined. The major tenets of each cognitive belief domain and links to OCD are reviewed below.

## INFLATED RESPONSIBILITY AND THREAT ESTIMATION

Individuals with an inflated sense of responsibility and an overestimation of threat believe that it is their responsibility to prevent feared outcomes and are quick to inflate the likelihood of the feared outcome occurring (Obsessive Compulsive Cognitions Working Group, 1997, 2003, 2005; Salkovskis, 1985). This can result in rituals performed to minimize this possibility, which often leads to a reduction in distress and responsibility. Studies have found a heightened sense of responsibility for harm in individuals with intrusive cognitions (Freestone et al., 1997; Rhéaume et al., 1995; Salkovskis et al., 2000). Additionally, an increased sense of responsibility has been identified as one of the strongest predictors of obsessional symptoms and has been shown to impact the frequency of compulsive behavior (Bouchard et al., 1999; Lopatka & Rachman, 1995; Salkovskis et al., 2000; Shafran, 1997). Research on adult OCD has also found this cognitive domain to be associated with symptoms related to doubting/checking and being responsible for harm (Brakoulias et al., 2014; Wheaton et al., 2010).

In children, inflated responsibility has been associated with higher levels of OCD symptoms (Magnúsdóttir & Smári, 2004; Matthews et al., 2007) and adolescents with OCD report a higher sense of responsibility than adolescents with anxiety disorders (Libby et al., 2004). Inflated responsibility has also been shown to fully mediate the effect of thought-action fusion and partially mediate the effect of metacognitive beliefs on OCD symptoms (Magnúsdóttir & Smári, 2004; Matthews et al., 2007). Barrett and Healy (2003) found that children with OCD reported higher ratings of feelings of responsibility, specifically for OCD-relevant threats, compared to a nonclinical group; however, no significant differences in responsibility were found between the OCD group and the anxious control group or between any of the groups with regard to non-OCD-relevant stimuli. Taken together, there are somewhat inconsistent findings with regard to whether excessive responsibility is unique to OCD or represents a transdiagnostic factor involved in different pediatric emotional disorders. Importantly, no published studies have examined the role of inflated responsibility in relation to the known symptom heterogeneity of pediatric OCD. This is an important gap in the literature as it could help explain inconsistent findings.

## PERFECTIONISM AND INTOLERANCE OF UNCERTAINTY

Perfectionism is a well-known cognitive characteristic associated with OCD (Pacht, 1984). Individuals with excessive perfectionism believe that perfect and/or exact solutions are possible and necessary for every problem. Perfectionism has been described as a product of difficulty tolerating uncertainty, a belief that all mistakes are intolerable, or a desire to avoid criticism (Guidano & Liotti, 1983). As a result of this belief system, perfectionistic individuals often engage in checking, mental reviewing, and reassurance seeking (Gershuny & Sher, 1995).

With regard to OCD, high levels of perfectionism have been found in adults with OCD relative to community controls (Antony et al., 1998; Ladouceur et al., 1996; Obsessive Compulsive Cognitions Working Group, 2001). While some studies have also found higher levels of perfectionism among those with OCD compared to individuals with anxiety disorders (Mavissakalian et al., 1993), another study did not find this difference (Obsessive Compulsive Cognitions Working Group, 2001). Thus, it is unclear to what degree perfectionism is specific to OCD rather than common across OCD and anxiety disorders. Regarding the heterogeneity of adult OCD, research has shown that perfectionism is associated with OCD symptoms that revolve around symmetry and arranging (Brakoulias et al., 2014; Wheaton et al., 2010).

Only a few studies have examined perfectionism in children with OCD. Consistent with the adult literature, one study found that children with OCD, compared to a nonclinical group, had higher levels of concern for mistakes, which is one dimension of perfectionism; however, this difference was not seen in comparison with an anxious control group (Libby et al., 2004). Ye et al. (2008) found that perfectionistic beliefs, and particularly sensitivity to mistakes, accounted for significant variance in OCD in a sample of children with OCD. Individuals with OCD often have an elevated need for certainty to predict and control events (Makhlouf-Norris & Norris, 1973). This need for certainty may play a role in the development and maintenance of compulsive rituals, which is supported by a study that found that doubting/checking was the most central symptom dimension (i.e., most strongly related to all other forms of symptoms) in pediatric OCD (Cervin, Perrin, Olsson, Aspvall, et al., 2019). Further, individuals that endorse checking compulsions have been found to exhibit greater levels of intolerance for uncertainty (Tolin et al., 2003) and intolerance of uncertainty has been found to be significantly associated with repeating and checking rituals (Tolin et al., 2003). Research about this construct in pediatric anxiety broadly suggests that intolerance of uncertainty is positively associated with worry and social anxiety symptoms (Boelen et al., 2010), but additional research is needed to further examine and understand perfectionism and intolerance of uncertainty in pediatric OCD. To our knowledge, no studies have investigated perfectionism and intolerance of uncertainty in relation to the symptom dimensions of pediatric OCD.

## OVERIMPORTANCE AND CONTROL OF THOUGHTS

Thought-action fusion (TAF) is a belief that thinking about an unacceptable or distressing event makes it more likely to occur and that having an unacceptable thought is equivalent to carrying out the associated behavior (Shafran et al., 1996). Among individuals with OCD, TAF is more common than in nonclinical individuals. Relatedly, adults with OCD endorse higher likelihoods of being able to use their positive thoughts to prevent distressing events (Amir et al., 2001). Further, magical thinking (i.e., the belief that thoughts or other mental phenomena may cause or influence unrelated external events; for instance, a child with OCD might believe his performance in a video game could influence geopolitical events) is common in pediatric OCD (Storch et al., 2007). While there is limited research on overimportance and control of thoughts among pediatric populations, studies have found an association between TAF and OCD symptoms in community samples of children (Bolton et al., 2002; Matthews et al., 2007). TAF has also been associated with generalized anxiety symptoms (Muris et al., 2001). Additionally, when compared to a nonclinical comparison group, children with OCD reported significantly higher TAF (Barrett & Healy, 2003; Kadak et al., 2014; Libby et al., 2004); however, no significant differences were noted between children with OCD and the anxious comparison group (Barrett & Healy, 2003). A study that examined TAF across children, adolescents, and adults with OCD found no differences in TAF across ages (Farrell & Barrett, 2006). Thus, it is possible that this cognitive bias may be present from an early age in anxious children.

Given the significant attributions associated with these thoughts, they are often accompanied by efforts to control them. One such strategy is thought suppression, which consists of an individual attempting to push an intrusive thought out of awareness after experiencing it (Wegner, 1989). Notably, attempting to suppress thoughts represents a part of the core diagnostic schema of OCD (American Psychiatric Association, 2013) and leads to tension, discomfort, anxiety, and negative mood (Bouman, 2003; Corcoran & Woody, 2009; Purdon, 1999). Individuals may also experience a rebound effect (i.e., the thoughts return in greater frequency) following the attempted thought suppression.

Findings related to thought suppression and OCD are inconsistent (Abramowitz et al., 2001; Najmi et al., 2010). Individuals who experience OCD symptoms endorse higher levels of attempts to suppress thoughts than nonanxious control participants (Amir et al., 1997). A small to moderate rebound effect was found in a meta-analysis (Abramowitz et al., 2001). Although the meta-analysis did not identify any differences in the effect between clinical and nonclinical samples, it is of note that it only included one study of individuals with OCD.

Limited research has examined thought suppression in children or adolescents within the context of OCD symptoms, but Wegner and Zanakos (1994) found that adolescents with OCD had higher scores on a measure of thought suppression than a comparison group. This suggests that adolescents with OCD tended to suppress intrusive thoughts. While we are not aware of any studies investigating associations between thought suppression and symptom dimensions of pediatric OCD, research with adults has shown this domain to be associated with unacceptable/taboo thoughts (Brakoulias et al., 2014; Wheaton et al., 2010).

## DYSFUNCTIONAL METACOGNITIVE BELIEFS

The cognitive model involves metacognitive processing (i.e., thinking about one’s thinking), but metacognition is not typically an explicit component of the model. Metacognitive beliefs often include positive meta-worry (e.g., worrying may help me avoid unwanted events and solve problems), negative meta-worry (e.g., worrying may make me go crazy), believing that thoughts may lead to negative outcomes, and cognitive self-consciousness and (non)confidence (Bacow et al., 2009). Self-reported severity of dysfunctional metacognitive beliefs has been found to be positively associated with self-reported OCD symptoms in nonclinical children and adolescents (Esbjørn et al., 2013; Mather & Cartwright-Hatton, 2004) and with self-reported (but not clinician-rated) symptom severity among treatment-seeking youth with OCD (Rizvi et al., 2020). Associations between metacognitive beliefs and OCD symptoms have also been identified across the age span of children and adolescents with OCD (Rizvi et al., 2020), indicating that cognitive beliefs may be relevant irrespective of age. Overall, the small body of research on metacognitive beliefs and OCD suggests that metacognition may offer new insights into pediatric OCD. Again, we are not aware of any studies that have examined metacognitive beliefs in relation to the known symptom dimensions of pediatric OCD. This is an important gap in the literature, as metacognition may be uniquely related to certain symptoms, which could have implications for treatment.

## CURRENT STUDY

While the existing research suggests inflated levels of dysfunctional cognitive beliefs among youth with OCD, additional research is needed. Understanding the associations between cognitive beliefs and the major symptom dimensions of OCD symptoms is important, as it is reasonable to believe that beliefs may play different roles dependent upon symptom presentation. Such differences may have direct bearing on treatment and personalized interventions. Furthermore, while research supports the role of cognitive and metacognitive beliefs in the onset and maintenance of OCD in adults, there is a need to expand this area of literature to pediatric OCD. Thus, the aim of the current study was to examine whether cognitive and metacognitive beliefs were differentially associated with the major symptom dimensions of pediatric OCD and whether age affected these associations.

To this end, we examined how self-reported cognitive beliefs (i.e., inflated responsibility/threat estimation, perfectionism/intolerance of uncertainty, and over importance/control of thoughts) and metacognitive beliefs (i.e., positive meta-worry; negative meta-worry; cognitive monitoring; and superstition, punishment, and responsibility beliefs) were associated with self-reported severity of OCD across the symptom dimensions (i.e., doubting/checking, obsessing, washing, hoarding, ordering and neutralization). Prior research has shown that doubting/checking and obsessing correspond to the harm/responsibility/checking dimension and are underpinned by fear and anxiety. Thus, these symptom dimensions are compatible with a fear-centric view of OCD, which is the basis of the cognitive model of OCD. However, ordering and, to some extent, washing, which correspond to the symmetry and contamination dimensions, respectively, are underpinned by other motivational factors (Cervin, Perrin, Olsson, Claesdotter-Knutsson, et al., 2019; Cervin et al., 2020a, 2020b; Rosa-Alcazar et al., 2014). Informed by this previous work, the following hypotheses were tested:

1. All cognitive/metacognitive belief domains will be positively and uniquely associated with doubting/checking and obsessing.
2. No statistically significant associations between belief domains and ordering will emerge.
3. Associations between the belief domains and washing will be weaker than between the belief domains and doubting/checking and obsessing.

No directed hypothesis with respect to the neutralizing dimension was outlined, as this dimension does not clearly correspond to any of the major symptom dimensions of OCD (Bloch et al., 2008). Further, no directed hypothesis with respect to the hoarding dimension was outlined because it is unclear if hoarding as measured in this study can be considered a core OCD phenotype. Last, at present, the literature is inconsistent about whether or not cognitive beliefs are more strongly linked to OCD in adolescents compared to younger children (Farrell & Barrett, 2006; Rizvi et al., 2020). Therefore, we examined whether the associations between belief domains and symptom dimensions differed in children compared to adolescents.

## Material and Methods

### PARTICIPANTS

Participants included 137 treatment-seeking children and adolescents with OCD. The OCD diagnosis was confirmed using the structured diagnostic interview Kiddie Schedule for Affective Disorders and Schizophrenia (Kaufman et al., 1997). All participants in this study were recruited as part of a treatment trial of pediatric OCD, which examined d-cyclosersine augmentation of cognitive-behavioral therapy (Storch et al., 2016). All youth completed the self-report scales in the current study as part of a pretreatment assessment, which was the only timepoint used in the current study. The mean age of the participants was 12.28 years (*SD* = 3.02; range: 7–17 years) and about half (52%) were female. A vast majority were White (87%), 5% were African-American, 6% were Hispanic, and 2% were Asian. A majority of caregivers (58%) had at least college education. The mean score on the Children’s Yale-Brown Obsessive-Compulsive Scale (Scahill et al., 1997) for the sample was 25.29 (*SD* = 5.88; range: 16–37), indicating moderate to severe OCD. Not all participants in this study entered the treatment phase of the trial, but all fulfilled diagnostic criteria for OCD, with OCD being their primary or co-primary disorder, and all had a CY-BOCS score of 16 points or higher. Sociodemographic information and means and standard deviations for all study variables for the age-split samples are in Supplemental Table 1. The study was approved by the institutional review boards at the University of South Florida and Massachusetts General Hospital.

### MEASURES

#### Obsessive-Compulsive Inventory–Child Version (OCI-CV)

Symptom dimensions of OCD were assessed via the OCI-CV which is a 21-item self-report measure of OCD symptoms in youth across six dimensions: doubting/checking, obsessing, hoarding, washing, ordering and neutralizing (Foa et al., 2010). Participants responded to each item by marking how often they experience 21 distinct OCD symptoms (0 = *Never*; 1 = *Sometimes*; 2 = *Always*). Item scores are summed to create the dimensional scales and a total score. OCI-CV has demonstrated sound psychometric properties in a line of previous studies in clinical and nonclinical samples (Cervin, Perrin, Olsson, Aspvall, et al., 2019; Jones et al., 2013) and has been shown to discriminate between youths with OCD and those with tic and anxiety disorders (Aspvall et al., 2020). All dimensional scales have demonstrated adequate internal reliability in prior studies among youth with OCD with the exception of the neutralization scale (Aspvall et al., 2020; Jones et al., 2013). The dimensions of washing and ordering have been shown to strongly correspond to contamination/-cleaning and symmetry symptoms while doubting/checking and obsessing have been shown to more strongly correspond to harm/responsibility/checking (Cervin, Perrin, Olsson, Claesdotter-Knutsson, et al., 2019; Garcia-Delgar et al., 2016).

#### Obsessive Beliefs Questionnaire–Child Version (OBQ-CV)

OBQ-CV is a 44-item self-report measure of cognitive beliefs related to OCD across the dimensions of responsibility/threat estimation, perfectionism/uncertainty, and importance/control of thoughts (Coles et al., 2010). Participants responded to each item by marking to which degree they agree with statements related to cognitive beliefs (1 = *Disagree Very Much* to 5 = *Agree Very Much*). The dimensional scales of the OBQ-CV have evidenced high internal reliability (alphas from .82 to .94) and sound construct validity in prior studies (Coles et al., 2010; Wolters et al., 2011).

#### Metacognitions Questionnaire for Children (MCQ-C)

MCQ-C is a 24-item scale that assesses dysfunctional metacognitive beliefs across the following subscales: cognitive monitoring; positive meta-worry; negative meta-worry; and superstition, punishment, and responsibility beliefs (Bacow et al., 2009). Participants responded to each item by marking how much they generally agree with each statement on a 4-point scale ranging from 1 (*Do Not Agree*) to 4 (*Agree Very Much*). The total score on the MCQ-C, a sum of all items, was used in the current analyses as a general measure of metacognitive awareness and processes. Higher scores indicate higher levels of metacognition. Internal reliability of the total score ranges from adequate to good (alphas from .73 to .87; Myers et al., 2019). A systematic review of the measure has found that the total score is consistently related to a range of internalizing symptoms (Myers et al., 2019). While Smith and Hudson (2013) found significantly higher scores in the clinical than the nonclinical group, Bacow et al. (2009), who controlled for worry content, did not detect this difference. This highlights the need to control for anxiety when examining associations between cognitive beliefs and clinical symptoms.

#### Multidimensional Anxiety Scale for Children (MASC)

MASC is a 39-item measure that assesses anxiety across the dimensions of physical symptoms, harm avoidance, social anxiety and separation/panic. It also produces a total anxiety score. Participants responded to each item by marking how true each statement is for them on a 4-point Likert scale (0 = *Never True About Me* to 3 = *Often True About Me*). Only the total scale was used in the present study. This scale has demonstrated high internal reliability and test-retest reliability in previous studies (March et al., 1997; March et al., 1999) and was used to control for anxiety when examining the associations between cognitive beliefs and OCD dimensions.

### STATISTICAL ANALYSIS

The study hypotheses were tested within a structural equation modeling (SEM) framework. As the variables were not normally distributed, maximum likelihood estimation with robust standard errors and a scaled test statistic equal to the Yuan-Bentler test statistic was used. A subset of participants (*n* = 38; 27.7%) had missing data on four of the six OCI-CV scales (hoarding and neutralization were not missing for this subset of participants). Missingness was caused by a coding error when the questionnaire package was digitalized for the study. Thus, missingness was assumed to be missing completely at random, which was supported by no statistically significant differences for participants with and without missingness for age, sex, MCQ-C, any of the OBQ-CV dimensional scales, the hoarding and neutralization scales of OCI-CV or CY-BOCS total score. No missingness was present for the cognitive/metacognitive belief scales. Age was missing for one participant. MASC data were missing for three participants. Missingness was handled using full information maximum likelihood (FIML) estimation within each SEM model. FIML is unbiased when data is missing completely at random (Enders & Bandalos, 2001) but increases statistical power and hence the possibility to detect true associations.

We first ran separate models for each cognitive/metacognitive belief domain. To this end, we fitted a regression model within a SEM framework with the cognitive/metacognitive belief domain as the independent variable and the six OCI-CV symptom dimensions as dependent variables. Then, we ran a full model that included all the cognitive/metacognitive belief domains and the six OCI-CV symptom dimensions. Covariance among the OCI-CV dimensions were included in all models; however, covariance between hoarding, washing and obsessing were set to zero to benefit model identification. Importantly, these dimensions were not statistically significantly correlated at the zero-order level (see Table 1). An alpha level of .05 was used as an indicator of statistical significance in all models. The statistical analyses were conducted in R Studio version 1.3.959 using the R-package *lavaan*. To test the hypothesis that the belief domains would be more strongly associated with doubting/checking and obsessing than with washing, standardized beta values were compared using the R-package *bain*. Using this package, directed hypotheses of parameters in a SEM model were tested within a Bayesian framework by computing a Bayes factor indicating the degree of evidence in favor of the hypothesis. A Bayes factor between 3 and 10 was interpreted as moderate evidence in favor of the hypothesis. A Bayes factor > 10 was interpreted as strong evidence in favor of the hypothesis. We also tested for possible difficulties with multicollinearity (i.e., highly correlated independent variables) by examining tolerance and the variance inflation factor (VIF). Tolerance values below 0.2 and VIF values above 10 were considered indicative of problematic multicollinearity. In order to compare results between children and adolescents, we split the sample according to age (<13 years and 13-17 years) and carried out separate SEM models using the two samples. The age split mirrored the child-adolescent division and was statistically beneficial, as two equal sample sizes emerged (*n* = 68 in both; age for one participant was missing). Last, to further examine the contribution of each cognitive variable to variation in symptom dimensions, we used dominance analysis which is a statistical technique in which all combinations of a set of independent variables are tested in relation to the dependent variable. This is useful when multiple correlated independent variables are analyzed. The full statistical script is included as a Supplementary material.

## Results

**FIGURE 1:**
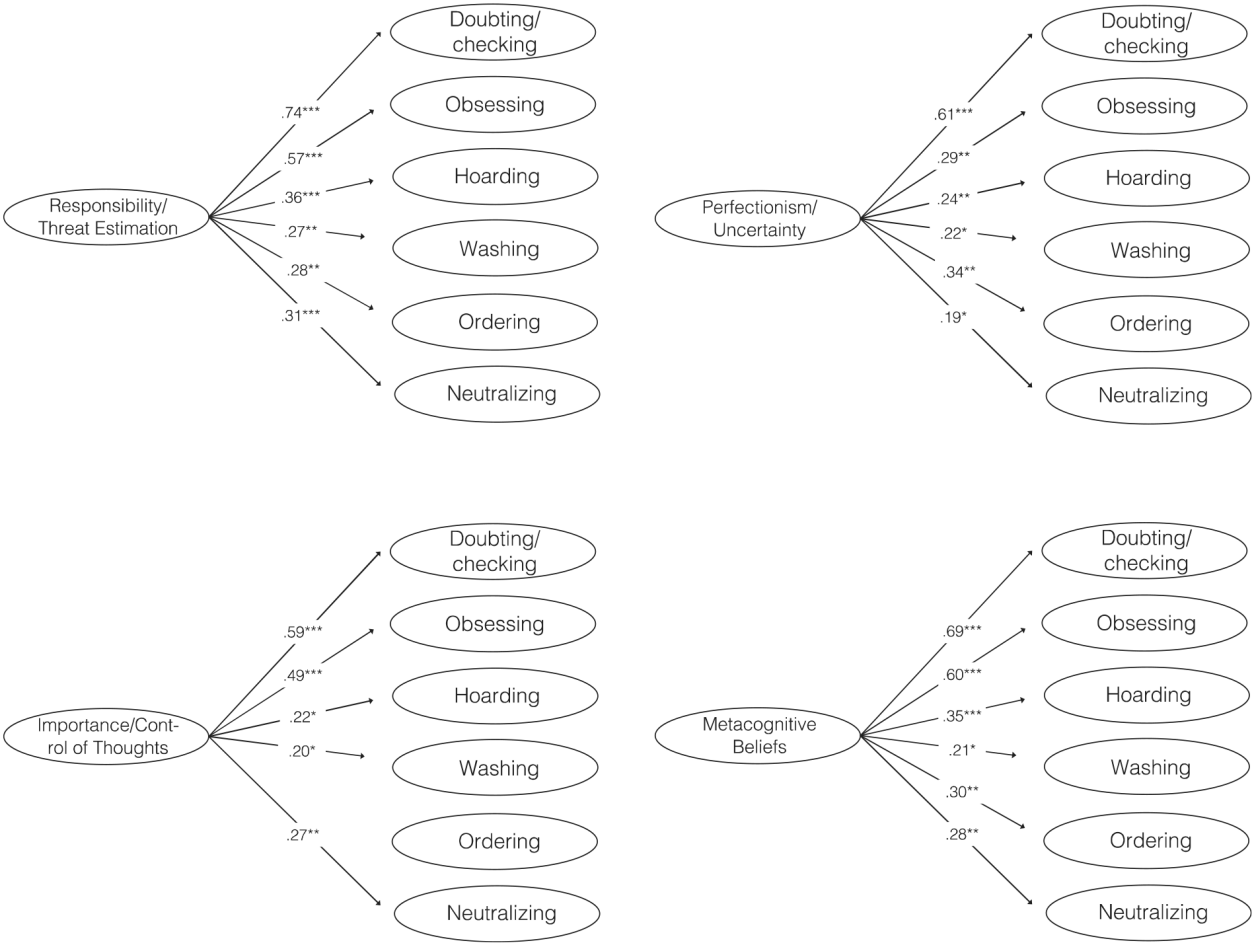
Associations (standardized regression coefficients) between each of the belief domains and symptom dimensions of pediatric OCD. *Notes.* * indicates *p* < .05. ** indicates *p* <.01. *** indicates *p* <.001.

Zero-order Pearson correlations and means and standard deviations for all study variables are in Table 1. Models with each belief domain as a separate independent variable are presented in Figure 1. Positive associations between each belief domain and all symptom dimensions were present for most belief domains, with the largest associations emerging in relation to doubting/checking and obsessing. We inspected tolerance and VIF values for the cognitive belief variables. No VIF value was above 4 and no tolerance value below 0.2 indicating that there were no problems with multicollinearity.

**FIGURE 2:**
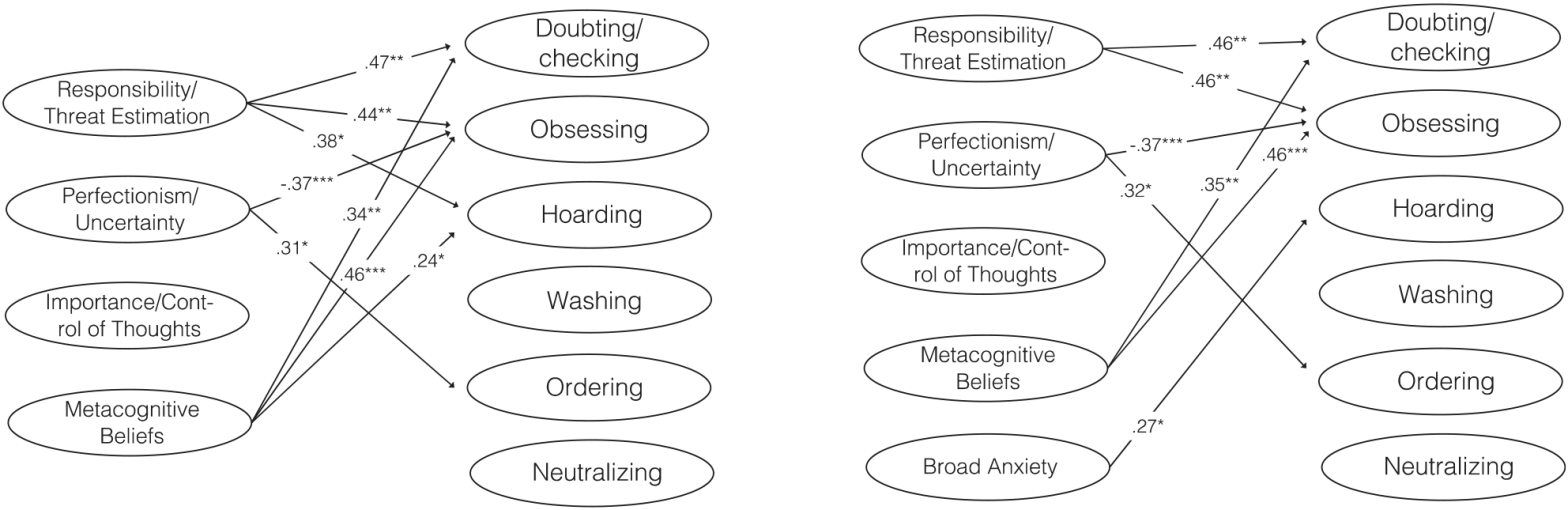
Unique associations (standardized regression coefficients) between belief domains and symptom dimensions of pediatric OCD. To the left, only belief domains are included. To the right, a variable capturing broad anxiety is included as a covariate. *Notes.* * indicates *p* < .05. ** indicates *p* < .01. *** indicates *p* < .001.

Figure 2 presents (a) a full model that includes all belief domains combined in a single model and (b) a model where broad anxiety is included as a covariate. Model/data fit for both models was good (Comparative Fit Index [CFI] = .987 & .985, Standardized Root Mean Square Residual [SRMR] = .026 & .025, Root Mean Square Error of Approximation [RMSEA] = .076 & .081; but with poorer Tucker Lewis Index [TLI = .874 & .832]; however, the *X*^2^ was not statistically significant in any model, indicating adequate fit). Responsibility/threat estimation and metacognitive beliefs were positively associated with the doubting/checking, obsessing and hoarding domains when broad anxiety was not controlled for and positively associated with doubting/checking and obsessing in the model that controlled for broad anxiety. Perfectionism/uncertainty was positively associated with ordering and negatively associated with obsessing in both models. Importance/control of thoughts was not significantly associated with any dimension in any of the models. No significant associations between belief domains and the washing and neutralizing dimensions were present in any of the models. Broad anxiety was positively associated with hoarding. Results from a model where broad anxiety was entered as a dependent variable along with the OCD symptom dimensions are presented in Supplemental Figure 1. Inflated responsibility/threat estimation and metacognitive beliefs were significantly associated with anxiety in this model (standardized regression coefficients of 0.41 and 0.34, respectively) and the associations between belief domains and OCD dimensions were nearly identical.

Our hypothesis that the belief domains would be more strongly associated with the doubting/checking and obsessing dimensions than with the washing dimension was tested within the Bayesian framework presented above. Moderate evidence was found to support that responsibility/threat estimation was more strongly associated with doubting/checking (Bayes factor = 7.90) and obsessing (Bayes factor = 4.47) than with washing. Strong evidence was found to support that the metacognitive beliefs domain was more strongly associated with doubting/checking (Bayes factor = 38.96) and obsessing (Bayes factor = 59.75) than with washing. Weak evidence was found in support of the hypothesis that the perfectionism/uncertainty and importance/control of thoughts domains were more strongly associated with doubting/checking and obsessing than with washing (Bayes factors: 0.02 to 2.34).

**FIGURE 3:**
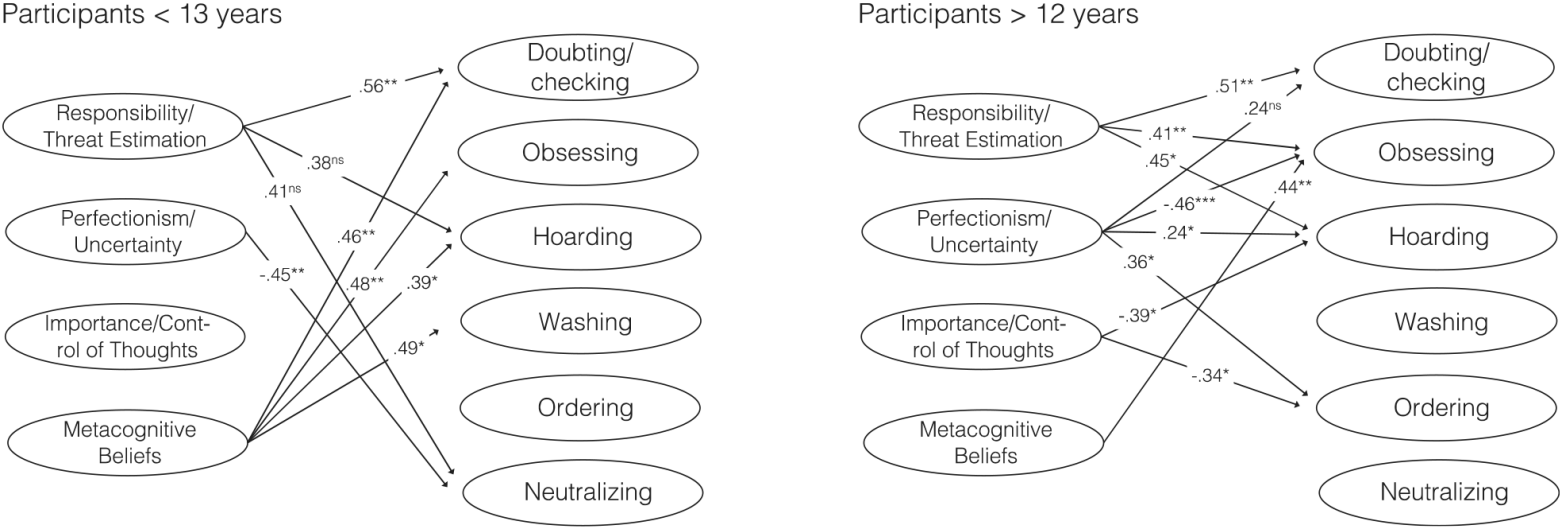
Unique associations between belief domains and symptom dimensions of pediatric OCD for participants < 13 years (*n* = 68) and participants > 12 years (*n* = 68). Because of low statistical power and to facilitate comparisons with the full sample, standardized beta values with *p* < .10 are included. *Notes.* ns = Not statistically significant (*p* < .10). * indicates *p* < .05. ** indicates *p* < .01. *** indicates *p* < .001.

Figure 3 displays separate models for children and adolescents. Responsibility/threat estimation was positively associated with the doubting/checking dimension in both models. Dysfunctional metacognitive beliefs were positively associated with obsessing in both models and among younger participants also with doubting/checking, hoarding, and washing.

Before conducting dominance analysis, we ran multivariable linear regression models with each symptom dimension as the dependent variable and the belief domains as independent variables. The following proportion of variance (adjusted *R^2^*) was explained for each symptom dimension: doubting/checking (61%), obsessing (46%), ordering (15%), hoarding (14%), neutralization (8%), and washing (3%). The average contribution of each belief domain in relation to each symptom dimension was then calculated using dominance analysis, and results are presented in Table 2. Inflated responsibility/threat estimation and dysfunctional metacognitions were the two belief domains that made the highest contributions to variation in each symptom dimension, although it was only in relation to doubting/checking and obsessing that they made substantive contributions.

## Discussion

In this study, we showed that dysfunctional cognitive and metacognitive beliefs play important roles in pediatric OCD, consistent with research in adults (Purdon & Clark, 1999; Rachman, 1997; Salkovskis, 1985). When shared variance among different cognitive beliefs was accounted for, the clearest links emerged between the belief domains of inflated responsibility/threat estimation and dysfunctional metacognitive beliefs and symptoms of doubting/checking and obsessing (i.e., intrusive thoughts). These associations remained after controlling for broad anxiety and were significantly stronger than the associations between the same belief domains and contamination/washing. Further, the belief domains accounted for 61% of variation in doubting/checking and 46% in obsessing, which should be compared to 15% for ordering, 14% for hoarding, 8% for neutralization, and 3% for washing. Thus, the belief domains of the cognitive model of OCD plus the addition of dysfunctional metacognitions appear to be substantially related only to a subset of symptoms in pediatric OCD. The present findings lay the foundation for future work to examine whether treatment for young individuals with symptoms that revolve around doubt, checking behaviors, and intrusive thoughts can be improved by incorporating interventions that target cognitive beliefs. We show that metacognitive beliefs may be as important as the belief domains originally included in the cognitive model of OCD, of which inflated responsibility/threat estimation appears to play a significant role. Incorporating cognitive interventions into treatment has been done successfully in adults (Wilhelm et al., 2009). Similar treatments have also been tested for youth with OCD by dedicating portions of treatment to change how obsessions are interpreted (e.g., by emphasizing how thought-action fusion and an inflated sense of responsibility lead to misinterpretation of intrusive thoughts that in turn triggers compulsions and thought suppression) (Bolton et al., 2011). Reinforcing learning after exposure and response prevention may be another way of targeting these cognitive domains in youth with OCD (McGuire & Storch, 2019). For instance, youth with OCD may misappraise the level of threat from an exposure. A clinician would establish the feared expectation and complete the exposure until the misappraisal feared expectation was disconfirmed. Afterward, the clinician could process the exposure to solidify learning and recalibrate threat estimations and feared expectations. Although the inclusion of theory-driven cognitive interventions is worthy of exploration, it is by no means certain that cognitive techniques work better than pure ERP for youth that present with dysfunctional cognitive beliefs. Dismantling studies that examine whether the inclusion of cognitive interventions moderates the effect that ERP has on both OCD symptoms and cognitive beliefs can help answer this question. It is also of relevance to explore whether interventions should target more narrow belief domains (e.g., inflated responsibility/threat estimation and dysfunctional metacognitive beliefs) or whether narrower belief domains reflect a broader cognitive tendency (which is somewhat indicated by the moderate to strong zero-order correlations between all belief domains in the present study) that perhaps can be addressed using less specific techniques or interventions.

Importantly, symptoms within the doubting/checking and obsessing dimensions correspond to only one of the three major symptom dimensions of pediatric OCD (when excluding hoarding), with contamination/washing and symmetry/ordering being the other dimensions (Bloch et al., 2008). In this study, contamination and symmetry OCD were not all or only weakly associated with cognitive beliefs when shared variance among both cognitive beliefs and OCD dimensions was accounted for. The exception was an association between perfectionism/uncertainty and ordering/symmetry. Dominance analysis showed similar results with each of the cognitive belief domains explaining only 1–3% of variation in washing and 3–9% of variation in ordering. Exposure therapy for OCD carried out in the 1970s and 1980s relied heavily on conditioning principles (Bolton et al., 2011). During the 1990s, cognitive models of OCD became increasingly popular. At present, standard CBT for pediatric OCD often includes exposure as a primary element together with cognitive interventions, but the emphasis of each component varies across treatments. In light of the present results, it is reasonable to ask whether cognitive interventions based on a fear-centric understanding of OCD should be included for all patients or only for the proportion of patients that actually experience difficulties with dysfunctional cognitive beliefs of this nature. Some studies suggest that pediatric patients with intrusive thoughts and checking compulsions may have a better treatment response (McGuire et al., 2019; Storch et al., 2008). Thus, it is possible that the combination of exposure and cognitive modules in modern CBT protocols match this kind of symptom presentation best. This is in line with studies demonstrating that stronger beliefs about responsibility and harm may be associated with a greater benefit of cognitive- but not behavioral-based treatment for adults with OCD (Steketee et al., 2019). Taken together, our findings suggest that contamination and symmetry symptoms do not map clearly onto the central tenets of the cognitive model of OCD. It is possible that these symptoms are driven and maintained by dysfunction in other psychological mechanisms such as sensory processing, emotion generation/regulation or executive functioning, or by cognitive processes not assessed in the present study.

It has been suggested that processes captured by the importance/control of thoughts domain can explain OCD onset, such that individuals that overvalue thoughts and then try to suppress them will experience a rebound effect during which the frequency and intensity of these thoughts increase, giving rise to obsessions (Abramowitz et al., 2001). Therefore, it was unexpected that this domain was not uniquely associated (i.e., when controlling for other cognitive domains) with any of the symptom dimensions of OCD. If replicated, this may suggest that importance/control of thoughts are not as salient as hypothesized for some children and adolescents with OCD, at least not in relation to the symptoms assessed in the present study or after symptom-onset has occurred. It is also possible, given the substantial correlations between importance/control of thoughts and other cognitive beliefs, that this thought process is used in response to other dysfunctional cognitive processes. Further, in the adolescent-specific analysis, negative associations between this domain and hoarding and ordering emerged which may suggest that patients who rely on behavioral compulsions (e.g., ordering) may prioritize behavioral or sensory feedback over cognitive processes to reach a sense of control.

To the best of our knowledge, this is the first study to examine associations between cognitive beliefs and symptom dimensions of pediatric OCD making comparisons to prior literature difficult. However, a similar study in adults with OCD showed that responsibility/threat estimation was linked to the contamination/washing dimension (Wheaton et al., 2010). This is in contrast with our results where no association between any belief domain and contamination/washing emerged and belief domains only explained 3% of the variation in the washing dimension. Implications may be that contamination/washing symptoms are underpinned by partly different mechanisms in children than in and adults. However, another study with adults showed results in line with ours (Brakoulias et al., 2014). Like previous adult studies (Brakoulias et al., 2014; Wheaton et al., 2010), perfectionism/certainty was associated with symmetry/ordering and responsibility/threat estimation with harm/checking. This suggests that for these dimensions, partly similar cognitive mechanisms may be in play across the age span. Studies that include both pediatric and adult patients are much needed.

It has been debated to what degree dysfunctional metacognitions may be relevant to OCD in younger children, as the cognitive abilities needed for this type of cognitive activity are not fully developed in this age group (Matthews et al., 2007). Therefore, we tested whether associations between belief domains and symptom dimensions were similar in children and adolescents. Overall, largely similar results emerged, as responsibility/threat estimation and metacognitive beliefs were associated with doubting/checking and metacognition with obsessing in both age groups. An unexpected finding was that metacognitive beliefs appeared to be more strongly associated with a line of OCD dimensions in children compared to adolescents. Specifically, among children, metacognition was associated with hoarding and washing in addition to doubting/checking and obsessing. This is worthy of further investigation and future studies should examine whether the content of the metacognitive scale is correctly understood by younger children. Findings also showed that metacognitive beliefs as captured by MCQ-C emerged as more relevant to pediatric OCD than metacognitive beliefs captured by the OBQ-CV domain of importance/control of thoughts. The MCQ-C is a broad measure that includes several aspects of dysfunctional metacognitive beliefs such as positive and negative meta-worry and cognitive monitoring (Bacow et al., 2009). Future work may want to examine whether some of these aspects are more closely related to OCD than others. Further, the finding that MCQ-C was strongly associated with different OCD dimensions in younger children suggests that future studies about (meta)cognitive processes in pediatric OCD should not limit themselves to adolescents.

Several limitations merit mentioning. First, OCD dimensions as measured in the present study do not perfectly correspond to the dimensions outlined in the only meta-analysis to date (Bloch et al., 2008). Future research should examine dysfunctional beliefs in relation to measures that more closely correspond to these dimensions. Additionally, interview-based measures of OCD dimensions would be preferable. Second, the cross-sectional nature of the data precludes any causal inference and although cognitive beliefs are theorized to precede symptoms, longitudinal studies, probably using intensive data collection, are needed to test any causal hypotheses. Studies examining the role of cognitive beliefs in relation to symptom change during treatment are also important and can help inform personalized interventions. Third, although we did control for broad anxiety, other variables (e.g., emotion generation/regulation, distress tolerance, cognitive control) related to both cognitive beliefs and OCD dimensions may contribute to the identified associations between beliefs and symptom dimensions in this study. Future studies should carefully consider which variables to include during study design. Fourth, few participants were from ethnic minorities, which may affect generalizability of results. Last, we had low statistical power in the age-specific models and these results should be interpreted with caution.

Dysfunctional cognitive and metacognitive beliefs appear to be highly relevant to pediatric OCD. However, responsibility/threat estimation and metacognitive beliefs stood out as most important and only in relation to certain symptoms (i.e., doubting/checking and obsessing). Future studies are warranted to examine whether targeting metacognition and inflated responsibility and threat estimation leads to better outcomes for youth with OCD who have symptoms that revolve around responsibility, harm, intrusive thoughts and checking. Similar work has recently been carried out with adults (Steketee et al., 2019), and methodological designs from that body of literature can inform studies with youth. The cognitive domains studied here were not readily associated with symmetry- and contamination-related OCD, and future work should try to outline mechanisms important to the onset and maintenance of these symptom dimensions in youth with the goal to maximize treatment outcomes.

## Supplementary Material

supplementary

## Figures and Tables

**Table 1:** Means, Standard Deviations, and Correlations of Study Variables

Variable	*M*	*SD*	1	2	3	4	5	6	7	8	9
1. OBQ-CV Responsibility/Threat	40.49	15.10									
2. OBQ-CV Perfect./Uncertainty	43.14	17.27	.70**								
3. OBQ-CV Imp./Control of Thoughts	28.20	12.03	.78**	.65**							
4. MCQ-C Metacognitions	47.65	13.85	.70**	.59**	.65**						
5. OCI-CV Doubting/Checking	39.19	30.86	.75**	.64**	.62**	.71**					
6. OCI-CV Obsessing	44.44	33.61	.58**	.32**	.52**	.62**	.51**				
7. OCI-CV Hoarding	30.41	30.84	.36**	.24**	.22*	.35**	.35**	.13			
8. OCI-CV Washing	44.22	38.30	.26**	.22*	.21*	.21*	.23*	.01	.15		
9. OCI-CV Ordering	41.25	33.70	.30**	.39**	.20*	.33**	.41**	.19	.34**	.30**	
10. OCI-CV	27.70	27.98	.31**	.19*	.27**	.28**	.45**	.26*	.30**	.04	.48**

*Notes.* Scores on the OCI-CV dimensions have been standardized to a scale of 0 to 100 to facilitate comparisons across the dimensions. *M* and *SD* are used to represent mean and standard deviation, respectively. OCI-CV = Obsessive-Compulsive Inventory – Child Version. OBQ-CV = Obsessive Beliefs Questionnaire – Child Version. MCQ-C = Metacognitions Questionnaire for Children.

* *p* < .05.

** *p* < .01.

**Table 2:** Average Contribution for the Belief Domains to Variance in Each Symptom Dimension According to Dominance Analysis

	Doubting/ ch. Adjusted *R^2^*: 61%	Obsessing Adjusted *R^2^*: 46%	Hoarding Adjusted *R^2^*: 14%	Washing Adjusted *R^2^*: 3%	Ordering Adjusted *R^2^*: 15%	Neutralization Adjusted *R^2^*: 8%
Inflated responsibility – Threat estimation	21%	19%	7%	3%	9%	4%
Perfectionism – Intolerance of uncertainty	12%	9%	2%	1%	3%	2%
Importance/control of thoughts	11%	5%	2%	1%	2%	1%
Dysfunctional metacognitions	19%	15%	6%	2%	5%	3%
