# Supplementary material for: Cognitive Beliefs Across the Symptom Dimensions of Pediatric Obsessive-Compulsive Disorder: Type of Symptom Matters
Source: Behav Ther. Author manuscript; Available in PMC 2022 Aug 23. (PMC9397538; doi:10.1016/j.beth.2021.08.001)
Supplement: supplementary [file NIHMS1829693-supplement-supplementary.docx]

**Supplemental Table 1.** Sociodemographic characteristics and mean scores on study variables in the full sample and age-divided subsamples. Scores on the OCI-CV dimensions have been standardized to a scale of 0 to 100 to facilitate comparisons across the dimensions.

|  | Full sample | Children (< 13 years) | Adolescents (13-17 years) |
| --- | --- | --- | --- |
| *n* | 137 | 68 | 68 |
| Age, *M* (*SD*) | 12.28 (3.02) | 9.71 (1.73) | 14.85 (1.40) |
| Females, *n* (%) | 71 (52%) | 34 (50%) | 37 (54%) |
| CY-BOCS, *M* (*SD*) | 25.29 (5.88) | 25.09 (6.30) | 25.46 (5.51) |
| OCI-CV Doubting/Checking | 39.19 (30.86) | 27.95 (24.26) | 48.81 (32.78) |
| OCI-CV  Obsessing | 44.44 (33.61) | 33.24 (30.49) | 53.41 (35.55) |
| OCI-CV  Hoarding | 30.41 (30.84) | 32.11 (30.52) | 29.17 (31.33) |
| OCI-CV  Washing | 44.22 (38.30) | 38.26 (37.28) | 49.07 (38.77) |
| OCI-CV  Ordering | 41.25 (33.70) | 30.68 (28.29) | 49.70 (35.50) |
| OCI-CV  Neutralizing | 27.70 (27.98) | 22.64 (25.41) | 33.09 (29.58) |
| OBQ-CV  Responsibility/Threat | 40.49 (15.10) | 36.82 (13.97) | 44.53 (15.08) |
| OBQ-CV  Perfect./Uncertainty | 43.14 (17.27) | 38.81 (15.53) | 47.87 (17.65) |
| OBQ-CV  Imp./Control of Thoughts | 28.20 (12.03) | 25.99 (10.03) | 30.65 (13.36) |
| MCQ-C  Metacognitions | 47.65 (13.85) | 43.19 (11.23) | 52.46 (14.54) |

*Notes*. CY-BOCS = Children’s Yale-Brown Obsessive Compulsive Scale. OCI-CV = Obsessive-Compulsive Inventory – Child Version. OBQ-CV = Obsessive Beliefs Questionnaire – Child Version. MCQ-C = Metacognitions Questionnaire for Children.

**Supplemental Figure 1.** Associations between belief domains and OCD symptom dimensions plus broad anxiety.

*Notes*. * indicates p < .05. ** indicates p < .01. *** indicates p < .001.
